# Transactions of the Medical and Physical Society of Bombay. No. V

**Published:** 1844-07-01

**Authors:** 


### TRANSACTIONS OF THE MEDICAL AND PHYSICAL SOCIETY OF BOMBAY. No. V.

THERE is a self-asserting power in scientific investigation, which, however long kept in abeyance, is sure, at length, to develop itself, some time or other. It may be kept depressed for many years by a variety of counteracting circumstances that would almost seem to indicate its non-existence, until, rising superior to conventional neglect, or official contempt, it forces itself upon the notice of the public. In no country has this been more remarkably the case than in India, where the spirit of research and improvement, unless it could be shown at once to have a cogent reference to revenue, has received but a very negative kind of encouragement from the State. That a field so extensive, so varied in character, and so fruitful withal, should have remained so long uncultivated, and have contributed, comparatively with its resources, so little until a very recent date, to the granaries of professional knowledge, may be a matter of regret, but ought not to be one of surprise. A tenure founded on trading monopoly, in no case can be considered as friendly to literature and science for their own sake. The spirit of its sway is not a liberal one, but, on the contrary, one opposed to such speculations on the score of expense. Nothing is admitted as laudable that does not furnish a conclusive array of figures on the profit side of the balance-sheet. "I hate Boetry and Bainting,"—said the first "wie German Lairdie"—who succeeded the Stuart Line on the English throne. His Majesty, the Georgius primus of that ilk, disliked what ran counter to his usual wie German lairdyism of ideas, and this is precisely the case with the counting-house genius, when it is invested with the truncheon of dominion.

Our Rulers in the East, for a long time, tried to manage an empire as if it were a shop in which some secret process of manufacture was going on, which the patentee dreaded an invasion of. A restless jealousy was continually in operation, lest "outside barbarians" should pry too narrowly into what was going on "over the way." A morbid anxiety was ever betraying itself, lest undue attention should be attracted even to the locality of the shop, (which distributed its wares to distant customers by agents of its own,) for fear some rival interest should set up another. Nay, there was even an unwillingness to let those who once entered the shop as operatives, out again in a hurry, lest they might, by their gossip, or influence, be the means of directing too much of the public attention to the said emporium. To turn the present to the best possible account and to let the future shift for itself, was the general rule. The dominant class had too much to do, to provide good investments for their masters (not forgetting themselves), to think of posterity, or to dream of fostering science and letters. At length, however, (for such things will happen) a taste for these, in the abstract, sprung up from the midst of bales and *purwannahs*, and the ASIATIC SOCIETY of Bengal became a centre of archeological, mystic, and statistical information. Here judges, and philological civilians, and field officers of observant or scribbling habits, delighted to exhibit their callow plumage, in days when, to appear in print was not the vulgar thing of course it is now, perhaps, considered. The thing had a smack of aristocraticism, and there was a certain savour of dignity about it, while all kept up the ball with a grave sedateness that made it the more delicious *desipere in loco.* A stray Doctor Sahib, was occasionally caught, who, by his learned lore, gave great zest to the whole affair, and was frequently the social steel whose collision struck sparks of lore from the silicious, measured and dried-up philosophers around him. Members of the medical profession, as a class, have nevertheless never been a popular or favoured one in India, nor are they, most probably, so anywhere. In general they were, and are, considered a necessary evil, an indispensable annoyance, to whom, or which, the rationale of reward was to be doled out with rather a niggardly hand ; and who, as respected either the Civil or Military classes, were deemed with, but not of them. The government never dreamed of consulting them, individually or collectively, no more than it would of taking the advice of their Lascars. They were not supposed to know anything beyond a pill-box range of intellectual acquisition. It was considered proper and decent to maintain them as a compulsory sort of establishment for fear of conventional and legal reproach. They were looked upon as a kind of mechanical or gastric necessity, and, to this very day, the type of the Company's policy towards their medical officers may be pithily recognised in some Skipper's advertisement, who informs the world, that the admirable craft he commands, will leave Blackwall, or the Downs, by a specified day, and clinches the attractiveness of her merits, by adding that—" she carries a Cow and an experienced Surgeon."

To Apollo alone must be left the boast—

Opifer per Orbem dicor.

To be hailed as *Opifer* anywhere, one must have something of a genial sphere of action. There must be an audience, before there can be the award of applause for " the poor actor who struts and frets his hour upon the stage."

While we acknowledge how little, for a long time, was done for medicine by medical men in the East, we keep in mind, and beg of others not to forget, the difficulties they had to bear up against. There was no stimulus whatever in the Department itself, and, consequently, all who could do so, made it a point to cut it as soon as possible and get under the sunny side of something better, and almost anything else was better than the medical service. Occasionally when the State, in the Commissariat or lower grades of the political department, might require the services of a tolerably clever fellow, he was borrowed (when no better could be found elsewhere) from the medical corps ; and his energies became from that hour lost in the walk of his profession for ever. He thenceforward turned his back upon it with disgust, glad to be well rid of a calling in which honor and profit were upon a parity of foundation, and both so low as to be out of sight of common spectators. Others, seeing that the possession of rupees was the veritable Aladdin's lamp, and that, in the capacity of medical drudges to military establishments, the attainment of wealth could only be looked for ad Græcas Kalendas, became a sort of bastard merchants, or underwent some other novel metempsychosis, changing into Indigo Planter, or any other form of being that gave a riddance from that long alley without turning, from which not a single solitary vista opened to ambition.

In spite, however, of all discouragements, the scornful neglect of superiors, and the leaden apathy of equals; now and then, and far between, a few might venture to shew the genuine Esculapian inspiration, and that their talk was not wholly of bullocks, (Commissariat, perhaps?) by directing themselves to the languishing interests of their profession. Some even ventured to publish the results of their experience. Such, however, were caviare to the multitude, and perhaps in the end would be out of pocket by the venture, at which the drones of the hive rejoiced, deeming that it served them right. The very circumstance of possessing a superior understanding might, at times, owing to the unfortunate concatenation of circumstances, be rather prejudicial than otherwise to medical officers, unless they were perfectly cognizant of the arcana of "*booing*," as laid down, in a very edifying manner, in a certain ethical treatise of a dramatic form, called "The Man of the World." But then, was there not the Medical Board, the natural head of the Medical Staff? Yes, a head much like that of the snake which we occasionally see as an emblem of time, devouring its own tail. The Presidencies of Bengal, Madras, and Bombay, each had its snake devouring its own tail! These Boards in their own estimation did much. They did not exactly extract sunbeams from cucumbers, or convert cobwebs into silk; but then, in order to demonstrate to the Government that they were good for something, they laboriously bebustled themselves in a much ado about nothing circle of gallipot surveillance, and vexatious interference with their juniors, who not unfrequently were their superiors in all that constitutes a good medical officer, save and except the invaluable quality of seniority in the list! On the other hand, for the encouragement of true science, for fostering zeal, and exciting emulation, and the spirit of philosophical observation, and drawing radii of results to themselves as the grand professional centre of the Service—they had no more vocation than the sounding-board has to tune the keys of a musical instrument. You might hear of them in any annals, but those of the healing art, and its collateral branches. Excellent tiger hunters, cotton merchants, pig-stickers, horse-jockies, or hoarders of gold mohurs they might be, but in the vestibule of "Augur Apollo"—you never heard of them. To them, indeed, the god always appeared—

*Nube*, candentes humeros *amictus*, or, in other words, wrapped up in his cloak, so as not to be known to them.

It is a curious fact, but it is believed to be a fact notwithstanding, that with few exceptions, Medical Boards have always been found in hostile apposition to those most distinguished on the Medical Staff for talent, information, and independence of character. Possessing, fortunately for the Service at large, but little power, yet, of the little they did possess, they too often made a mean, invidious or contemptible use; merging their energies in petty objects, or paltry savings, which they mistook for economy. They took in its narrowest and most literal sense, what otherwise might be a lesson of true wisdom—

"Magnum Vectigal parsimonia,"

and thus compromised real efficiency, or worse still, the well-being of the sick and the weak. Thus it was that in Calcutta, during Lord William Bentinck's time, they being anxious to add a hue to the rose of his economy, at the expense only of their juniors, suggested to his Lordship, quietly from themselves, and a-propos to nothing, that the situation of Surgeon to the General Hospital might be abolished, even though it was held by one of his own personal staff, who was acknowledged to be an honor to the service. The Board further modestly suggested, that an Assistant-Surgeon might do the duty performed by the Surgeon, while the pay of the latter might thus be saved to the Government. His Lordship, who clearly saw through this liberal proposal, and thoroughly appreciated the purity of generous public spirit, no less than the taste and delicacy in which it was offered, is understood to have replied, that he did not deem it expedient, and that, with reference to an Assistant-Surgeon doing the duty, he might by parity of qualification, perform the duties of a member of the Medical Board also.

To the paralysing influence of the Medical Boards is partly, or in a great measure owing, the slow rate, for so many years, of medical progress in India. Occasionally, a man much above the average, a Gibb, an Annesley, or a Playfair, might get into the Board, but what could he do as a minority, or at any rate as a counterpoise against a radically bad system? The best men must have pined in such a position. To generous talent, and aspiring nobility of soul, it must have been crushing. Sickened by the withering sense of their own insignificance for purposes of amelioration, and real ineffectiveness with the Government for large and systematic good, (whatever might be professed by secretaries and officials in high places,) such men must have felt that it was useless to struggle against the stream. As a deliberative body, the Government has generally evinced total indifference to its suggestions, or its measures. Boards, indeed, have usually been considered a kind of paddock, where the worn-out hacks of the profession might graze out the remainder of an inglorious official existence in peace.

A dawn of better times broke upon the profession in the formation, some seventeen years ago, of the Medical and Physical Society of Calcutta, which has ever since continued a moving spring for eliciting information on a variety of topics of the greatest scientific interest; although at one time it was reduced, we hear, nearly to the verge of bankruptcy by injudicious management, and a party-spirit that, it is to be hoped, has been laid in the Red Sea for ever! That it is now flourishing again in a state of vigour, is in a great measure due to the tact, talents, and conciliatory spirit of its present accomplished and worthy Secretary, J. J. Jackson, Esq. In regard to the Medical Society of Calcutta, justice demands it to be acknowleged, that the members of the Medical Board, have always, so far as our information goes, shewn themselves its friends, and, as far as their power might go, its patrons. It is indeed pleasing to have any thing to read that redounds to their credit. O si sic omnia! For many years the Medical Officers of Madras and Bombay (including those of the Royal service) generously supported the Calcutta Association, enriching its annals with valuable contributions. But in process of time the other Presidencies instituted societies of their own upon similar principles, and all three are now in active operation. A great re-action had thus unquestionably taken place, which ever since has been deepening and widening its current, and may be expected to be further instrumental as a reviving power of improvement, provided the State also look to it, and facilitate by means of its own, at little real cost, the flow of its channels and feeding streams. Although in a region where previously a, comparatively speaking, Cimmerian darkness had prevailed, this enlightenment was auspiciously felt throughout the whole body of the profession in the East, still there was a void, which the appearance at long intervals, of an occasional volume of Transactions could not fill up. The want of a monthly medical periodical became palpable. In India serious difficulties beset the first establishment of a literary project of any kind. The merit of establishing the first medical periodical in India is due to Messrs. Grant and Pearson of the Bengal Presidency, who on the 1st January, 1834, at their own sole responsibility and cost, it appears, issued the first Number of "the India Journal of Medical Science," which was, a year or two afterwards, transferred to Mr. Corbyn, who has continued to conduct it with great zeal and success. Some years afterwards, under the able management of Mr. Rogers, a similar medical periodical was established at Madras; nor has Bombay been wanting in the good cause.

So far then, the members of the profession in India have done their part and done it well, all things considered. They have in some measure roused themselves out of the lethargic state in which they lay so long, through the soporific influences of causes that we have endeavoured to lay impartially before our readers. It remains now for the authorities to do their part, aye and to do it in a statesmanlike manner too. Of late they made a good beginning in ameliorating the scale of retiring pensions, so as to bring medical officers nearer to a par in that respect with their military brethren. Much, however, yet remains to be done before an approach even to moral equality of privileges and immunities can be made out on the part of the medical officer. The Medical Service as a body stands urgently in need of being raised in public estimation. Mere self-respect will not always support a corps unless it unmistakeably feels, and sees that others acknowledge it to be held in honor by the State. No such conviction can possibly support the Indian medical service, since we have it upon evidence which we deem conclusive that it does not exist. That Service feels itself, from the treatment it has received and continues to receive, to be *a low corps*. Nothing has yet been done by the State emphatically to evince the contrary. Mere emoluments and pensions will not do this, even were they still greater than they are. Something more than the easy means of animal and social sustentation is required here. The mind of man has its lacteals as well as his physical frame. The spirit has its chyle without which it pines and dies. If the Medical Staff of India be not considered a low corps at head quarters, unquestionably their systematic exclusion from all honors and distinctions whatsoever, on the part of the State, effectually brands them as such in the face of their military brethren. What mark of consideration has the Government ever conferred on distinguished officers of this class? In all other departments of the service, when the head of a department retires, or dies, some graceful mark is enunciated in the Gazette, of the sense entertained of his merits and services. Nothing of the sort is done as regards the heads of the medical department of the army. They may come or go, live or die, it is all the same, there is an exalted unconsciousness exhibited in the organs of public expression of their having ever existed. No notice, no compliment, no order, no honor of any kind ever awaits them living or dead. For this studied superciliousness and neglect there must surely be some cause ? What is it ? Or is it causeless and wholly unmerited ? Either they do not deserve it or they do—it is intentional or it is fortuitous. If the latter, it ought forthwith to be remedied, and large amends made for past exclusion ; if the former, then are they thus confessedly stamped as forming a class, to whom it would be derogatory on the part of the State to pay any public compliment whatsoever—for pay and allowances and pension are no compliment at all, but the quid pro quo of hard service and exile. On either horn of the dilemma, the cause of this scandalous neglect rests, and it may be added as a corollary on this " eternal fitness of things "—that in a country where the heads of the clergy are received with salvos of cannon, the heads of the Army Medical Department have no salute whatever accorded to them. We have stated that all the reward they have then, or ever can expect, is pecuniary. In all campaigns there is a lively excitement produced in every grade by the prospect of advancement and distinction. Medical officers unhappily are the exception. To them it is a very up-hill prospect indeed, all responsibility, painful anxiety and toil, but no chance whatever of advancement or distinction. They behold those who entered the army many years after them pass them in the race, honored and bedecorated, while they may go on from Burmah to Burtpore, from Ghuznee to Meeanee unnoticed and undistinguished. But they are in excellent company—look at the Royal Medical Service. Did any honour devolve on the surgeons who shared in the perils and toils of our great naval engagements ? Where are those that should have rendered effulgent the heads of the medical staff in the Peninsular campaigns ? But has not the head of the Army Medical Department been made a Baronet ? Indeed ! and have not Brewers and Stationers been created Baronets ? Why should not the head of a great department receive a Peerage as well as others who have not deserved a jot better. " Podalirius et Machaon secuti ducem Agamemnon a Trojano bello, attulerunt non mediocrem opem suis commilitionibus." Alas ! the great Duke deems that he has done enough for the Army Medical Staff. They have no medals, no escutcheons, no ribbons. Unlike Menelaus, His Grace never came under any Machaon's hands in the day of battle, nor did an army look, as in the case of the great Grecian leader, to one divine man as holding in his hands, while the life blood welled over his harness—the fate as it were of Greece. God forbid that the great Duke should ever need such aid. If he had needed it in time past, perhaps it were better for the Medical Staff. Be that however as it may, it will hereafter reflect no credit on his memory, let his flatterers say what they please, that when he might have honored science, and noble zeal, in the line of the most arduous of professions, he neglected it with the cold impassiveness of aristocratic scorn.

We have thus endeavoured fairly to shew cause why the field of Indian research has not been more productive. Nevertheless, however great the negligence of their superiors may have been to their just claims, we must remind the Indian Medical Service, that the genius of their noble profession will not hold them excusable, if they halt in any respect behind the spirit of the age. Considering how recent the re-action has been, and how little has been done for it by the State, the candid enquirer will allow that it is very honorable to that service. One of their own number has forcibly pointed out to them a walk in which much remains to be done, and in which he has himself been a distinguished labourer; we mean the field of Medical Statistics, in which Mr. J. R. Martin has so successfully led the way. " We may not," (says Hobbes*) " as in a circle, begin the handling of a science from what point we please. There is a certain clue of reason, whose beginning is in the dark, but by the benefit of whose conduct, we are led, as it were, by the hand, into the clearest light, so that the principle of dictation is to be taken from that darkness, and then the light to be carried thither for the irradiating its doubts. As often therefore as any writer doth weakly forsake that clue, or wilfully cut it asunder, he describes the first steps, not of his progress in science but of his wanderings from it." Is not this observation in relation to ethics eminently applicable to the subject of medical statistics as a clue to generalisation in tracing the history of disease and climate? We would strongly impress upon our brethren in India the excellence of the advice given to them by Mr. Martin—" let them seriously examine the influences of general tropical climate, of locality, and of season on European health; such objects are in reality of more value than volumes of cases, or details of routine practice; their careful investigation will confer permanent benefit on the public service, and sooner or later derive honor to themselves, difficult if not impossible to be obtained in any other way."† Let them turn up the same work in regard to the climate of Bengal, and from the motto of Cabanis (p. 15) to the running commentary upon his and Malte Brun's text, they will find an abstract of what is meant by climate felicitously illustrated throughout the whole work, the constant recollection of which will be edifying and profitable. The subject is by no means new. The following observations are two hundred years old.

" Lemnius reckons up two main things most profitable and most pernicious to our bodies: air and diet. Such as is the air, such be our spirits, and as our spirits, such are our humors. It offends commonly if it be too hot and dry, thick, fuliginous, cloudy, blustering, or a tempestuous air. Bodine, in his fifth book proves that hot countries are most troubled with melancholy. * * * * Cold air in the other extreme is almost as bad as hot. * * * The worst of the three is a thick, cloudy, misty, foggy air, or such as comes from fens, moorish grounds, lakes, muckhills, draughts, sinks, where any carcases or carrion lie, or from whence any stinking fulsome smell comes. * * * Alexandria, an haven town in the Mediterranean Sea, Saint John de Ulloa, an haven in Nova-hispania, are much condemned for a bad air, so is Durazzo in Albania, Lituania, Ditmarsh, Pomptinæ Paludes in Italy, the territories about Pisa, Ferrara, &c. Romney

---

* "Philosophical Rudiments."

† "Influence of Tropical Climates, &c." 6th Edition.

Marsh with us: the Hundreds in Essex, the Fens in Lincolnshire. * * * The best soil commonly yields the worst air, a dry sandy plat is fittest to build upon, and such as is rather hilly than plain. * * * Constantine *(de Agricult.)* praiseth mountains, hilly steep places, above the rest by the sea side, and such as look towards the North. * * * *Serenat Boreas,* the North wind clarifies, but near lakes or marshes, in holes, obscure places, or to the South and West, he utterly disproves. Those winds are unwholesome, putrifying, and make men subject to diseases. * * * Varro de re rusticâ forbids lakes and rivers, marsh and manured grounds, they cause a bad air, and gross diseases hard to be cured."*

Not only are diseases frequently changing their character, but modifications of condition produce new crimes, as well as new diseases. The last fact was well known to the ancients—" vero etiam nova genera morborum sæpe incidere in quibus usus ostenderit adhuc nihil."† The immense importance indeed of the neglect of Medical Statistics in all its extensive bearings, is so obvious, that we do not deem it necessary to dilate upon it further here, more especially as the attention of Indian medical officers would seem to have been aroused to it by the General Order of Nov. 1835, which originated with Mr. Martin. The work, the title of which heads these remarks, sufficiently indicates this gratifying fact, for almost all the items of the Number in question point in the right direction—as may be seen by a glance at the Table of CONTENTS :—

1. Report of the Diseases which have occurred in the Honorable Company's Steam Flotilla on the river Euphrates, from the 1st January, to the 31st December, 1841, together with an account of the Climate and Medical Topography of the Upper Euphrates. By John C. Floyd, Esq.—2. Annual Report of the Native General Hospital at Bombay, for the year 1841. By A. Graham, Esq.—3. Annual Medical Report of H. M. 2d or Queen's Royal Regiment, from the 1st of April, 1841, to the 31st of March, 1842. By R. H. A. Hunter, Esq.—4. Portion of the Annual Report of the 1st Troop Bombay Horse Artillery, embracing the first eleven months of the year 1841. By A. H. Leith, A.M.—5. Reports of the Medical Statistics of Upper Scinde, drawn up by the Medical Officers serving with the Force under the command of Brigadier England.—6. History of the Epidemic Fever which prevailed among the men of Her Majesty's 17th Regiment during the Monsoon of 1841, when quartered in the Colaba barracks, Bombay. By Arthur S. Thomson, M.D. Assistant Surgeon, 14th Dragoons, lately 17th Foot.—7. Annual Report of the 2nd Battalion, Bombay Artillery, stationed at Fort George, for the year 1841. By T. Robson, Esq.—8. A Letter addressed to the Superintending Surgeon of the Deccan Division, on the use of the Iodide of iron. By D. Grierson, Esq.—9. A Letter on the Fever which prevailed at Bankote in the S. Concan in 1841, addressed to the Superintending Surgeon of the Presidency Division. By T. Waller, Esq.—10. Remarks on the practice of Transfusion and its application in the treatment of Hæmorrhage, &c. deduced chiefly from Recorded cases. By John Peet, Esq.—11. Medico-

* Burton's Anatomy of Melancholy.

† Celsus, Lib. 1.

Historical Abstract of the 1st year's service in the East Indies, of H. M.'s 14th Regiment of Light Dragoons, at Kirkee, under the Bombay Presidency. By J. W. Moffat, Esq. Surgeon H. M.'s 14th Light Dragoons. —12. Observations on the good and bad effects of Calomel in some of the Diseases of India. By J. Murray, Esq.—13. Annual Report of the 25th Regiment, N. I. for the year 1841. By A. Wright, Esq.—14. Annual Report of the Civil Surgeon of Ahmedabad for the year 1841. By S. Sproule, Esq.—15. Contributions to Vegetable Embryology, from observations on the origin and development of the Embryo in Tropœolum Majus. By Herbert Giraud, M.D. Edin. F.B.S.E. Ext. Mem. Med. Soc. Edin. &c. &c.

APPENDIX.—1. Extract from a Letter on the Sickness at Kotra in Upper Sinde, addressed to the Surgeon, from W. T. Babington, Esq.—2. Table, shewing the comparative Means and Extremes of Rain-fall and Temperature, at Malcolm Peth, Paunchgunnee, Bombay, and Poona, during the Rainy Season of 1842. By J. Murray, Esq.—3. Annual return of Patients in the Lunatic Asylum, Bombay, from the 1st January to the 31st December 1841. By W. B. Barrington, Esq.—4. The late Assistant Surgeon John Fraser Heddle.

Though the details generally do not admit of being very conveniently quoted, they have the merit of being clearly and plainly drawn up, and to be free from that leaven of ambitious writing, which is never so much out of place as in medical records. They afford also a refreshing contrast to the old routine in giving, faithfully, features of physical appearance and peculiarity different from a fashion which is too prevalent when a man travels from Dan even to Beersheba, and his journal affords no testimony to the fact, save in symptoms of ailments.

Mr. Floyd's report refers to a line of country that the medical man's attention is seldom called to, in the way of his profession, though it is stereotyped as it were on the memory, in regard to the ancient history of many nations. The Valley of the Euphrates, from the 33rd to the 37th degree of N. lat. is for the most part hilly, and, except in the vicinity of Hilla and Bagdad, devoid of canals. It varies in breadth from two to five miles, except between Hilla and Anna, where it closes in, and becomes in many places less than half a mile in breadth. Second in importance to Hilla is the next station of Musseib, a place of considerable traffic containing about 5000 inhabitants. From this to Felugia, the banks are liable to be inundated during the rainy season. Felugia is in the same latitude as Bagdad, being 34 miles West of it. Here they proposed to establish a Hospital, in the event of the Euphrates being permanently available to us. From this to Hitt, the country begins to rise, a distance of 80 miles. Hitt is situated on an isolated bank of lime-stone rock, and has a population of 3000 souls, for the most part of a sickly aspect, which Mr. Floyd attributes to the impregnation of their atmosphere with sulphuretted hydrogen from the bituminous spings. Bitumen is the staple of the place, being used for a great variety of purposes. From this place to Deir is 252 miles as the river winds. The valley becomes broader, varying from 3 to 10 miles, with thick and extensive Tamarisk jungles. All through this tract, is desolate, which perhaps of itself may be conclusive as to its unhealthiness. From Deir to Jiaber Castle is 180 miles. The country becomes more elevated and interesting, "the banks of the river being lined in many places with mulberry and poplar in addition to the everlasting tamarisk: cultivation is also more extensive as we proceed northward." This place, which has been used as a station for the steamers, is situated on an isolated hill of sandstone and gypsum, nearly 30 feet above the level of the stream, which flows in a rich and beautiful plain, beneath its time-worn Saracenic ramparts. The valley here is about two miles broad. As the part of the river between Jiaber and Belis is on the direct road to Aleppo about 76 miles off, it has been proposed as the most northerly station. From Jiaber to Belis is 50 miles, the banks being well cultivated. From Belis to Birjeek, lat. 27° N. is 105 miles through a mountainous and desolate country. It contains 6000 inhabitants, and was the place selected for the erection of the steamers of the first Euphrates expedition. The climate, as is the case with all the northern parts of the Euphrates, comprises the extremes of heat and cold. It is distant from the Persian Gulf by river navigation 1,100 miles, 90 from Aleppo, by an excellent road, and 187 from Iskanderoon, the nearest port of the Mediterranean, and the place he thinks is admirably adapted for a depôt and for purposes of mercantile intercourse. Vegetation on the upper Euphrates is exceedingly scanty, the result no less of climate than of neglected irrigation. Immense jungles of tamarisk, willow, and liquorice, line the banks. The vegetable productions cultivated by the Arabs are wheat, barley, sesamum, &c. Melons, cucumbers, &c. grow to the greatest perfection. The seasons on the Upper Euphrates may be considered in every respect as much later than to the South. The Spring is cooler and more prolonged, and the great heat of Summer diminishes 20 per cent. compared with Bagdad. During the prevalence of the S.E. wind, headaches, diarrhœas and catarrhs are apt to supervene. Mr. Floyd, like all who have had personal experience of such oppressive winds, is convinced that their effect on the human system is owing to electric rather than atmospheric changes. Owing to the dryness of the soil and atmosphere miasmatic fevers do not prevail on the Upper Euphrates. Inflammatory fevers formed the largest and most serious class of disease, and occurred chiefly in June and September, when the diurnal range of temperature was the greatest, with cold raw mornings occasioned by the N.W. and Easterly winds. It comes on suddenly, and is characterised by great languor and soreness all over the back and extremities. This is succeeded by severe head-aches, flushed face, hot skin and restlessness—pulse 98 to 115, full and bounding. This state of excitement may be arrested in 24 hours by active antiphlogistic treatment. Though the climate of Mesopotamia is rather hot in Summer, it is free from endemic disease, and for the next nine months, Mr. Floyd states (which is no limited praise) that "there is not a finer climate in the *Universe.*" Why has the Editor deprived us of Mr. Floyd's cases? At any rate why not give us his sick return? Was he ashamed of it as a specimen of Medical Board Statistics?

Mr. Graham's annual report of the Native Hospital at Bombay shews an amount of mortality that tells as a sign of civilization. This sign, however, may be of double import, since the population of a country old in civilization, may, in the lower grades of society, evince a deteriorating or retrogressive condition. Natives of India (and it is pretty much the case elsewhere) do not enter the wards of an hospital until compelled by extreme suffering, when they are admitted in the last state of disease and destitution. Many in fact literally come to die. It is gratifying to learn, notwithstanding, that the deaths were rather more than two per cent. less than during the preceding year, which is attributed to the almost total absence of epidemic disease which had prevailed to a considerable extent the year before that, when cholera first, and small-pox afterwards, proved very destructive, as they had periodically done in preceding seasons. As respects the second of these scourges, how stands vaccination throughout India? Is it stationary, or progressing, or what? We ask these questions not without cogent reason. We hope that no false reasoning on the part of Government, or no suggested favour-currying stinginess on the part of the Medical Boards, has affected in any way the extensive efficiency of this invaluable prophylactic? From what reached us not long ago, regarding certain nibblings of the Bengal Board with the subject, we are not without apprehensions regarding it. One thing, from all we have heard, is clear—which is, that the scale of remuneration allowed on account of the duties is not sufficient. It has been calculated on a very niggardly basis. There has been too great a tendency of late years to reduce vaccine establishments in India, and to abstract from their popularity by rendering the duties almost compulsory on the lowest amount of pay—just as with medical attendants on poor-law establishments in this country. It seems, then, as if any where, any thing, were deemed good enough for medical men. Somehow they are more compressible by the State than the members of the other learned professions. How is this? Simply, we believe, because, as great corporations, the others have stood by each other as one man, and made themselves to be felt as "great facts," in the Institutions of the country, and have had accordingly representatives in the councils of the nation out of their own bodies, who, aided in legislating for them, and preserving their interests from encroachments of any kind; whereas, our wretched ill-assisted, ill-protected medical corporations, have never been so represented; but have had the beggarly shreds of legislation that scarcely cover their moral nakedness, provided by the haughty hands of the proud aristocracy, that regards them as the Gideonites of the learned professions. Until medical corporations have some degree of influence in the Senate through more direct representation, they will always be kicked about as the football of the minister of the day, and amenable to all sorts of back-stair trickery in regard to their rights and privileges, if it can be truly said that they have such! But to return to our mutton—small-pox is so terrific and loathsome a scourge, that all governments ought to consider its eradication or neutralization, a point of vital importance, and should be quick rather to listen to proposals for more extensively checking it than to specious suggestions for reducing establishments—because, perhaps, they may have been so effective in abating the ravages of the scourge, as to produce a lull that induces an idea that such may be dispensed with altogether. When Mr. Graham states that the year 1840 was remarkably free from any epidemic, we wish he had endeavoured to shew how this happened. It is all very well to give us returns of the numbers of sick relieved, and so forth, in diseases, but we desire something more in the way of inductive reasoning on the causes of such results. "The prevailing diseases," we are told, "were Cachexia, Diarrhœa, Dysentery, Fevers, *Fractures*, and Hepatic Affections."

Whatever the Medical Board may order in the way of returns, surely it is neither accurate nor scientific to return fractures and wounds under the head of diseases, and *prevailing* diseases too, just as if an easterly wind broke a man's bones or gashed him on the face! We cannot compliment our Reporter upon his account of post-mortem examinations in a case of Apoplexy; it is about as succinct and indefinite as the *no* history of his other apoplectic cases. Of Spasmodic Asthma, we are told in rather an off-hand way, that they were all successfully treated "on the usual plan, namely, by emetics, antispasmodics of ether, hyosciamus, *colchicum*, opium and conium, together with expectorants of ammoniacum, squills, tartarised antimony, and *digitalis*," &c. Here, to say nothing of colchicum figuring as an antispasmodic and digitalis as an expectorant, we have not the slightest guide to any particular mode of administration, nor is this looseness of record confined to this place. Under the head of cutaneous affections, we have had cases of Elephantiasis or Lepra Arabum, of which no hope of cure could be entertained. "All that could be effected towards their relief, consisted in the application of astringent lotions to the ulcerated parts." We should like to know the component parts of the said lotions. But, after all, so long as treatment of a constitutional disease, originating in probable pravity of the source of all secretion,* is confined to the local application of a lotion, it signifies little what it may have been composed of. Would it not have been worth while, in such cases, to have given a steady trial to the *Mudar?* Cachexia is an excellent witch's cauldron sort of class wherein to throw all diseases "that puzzle the will" to name them. Under this head, as here alluded to, not a single ray of light breaks upon us as to the precise nature of the ailments; which is the more perplexing, as Scurvy and Syphilitic Affections have a place of their own; and this admirable specimen of Medical Board Statistics winds up with another witch's cauldron under the designation of "*other diseases.*" Anomalous, is also a heading in such returns, so that the medical staff have a liberal choice of witch's cauldrons, as "anomalous," "cachexies," and "other diseases." This reminds one of the old chapter de omnibus rebus et *quibusdam aliis*. How is it possible to deduce correct statistical conclusions from returns thus bungling in their data?

Of five cases of Delirium Tremens that came under Mr. Graham's care, one proved fatal. One of the successful cases (an East Indian), who was greatly addicted to drinking, is instructive, as shewing not only the necessity for a heroic remedy, viz. opium, but the extent to which it must

---

* A-propos, at p. 31 we have the remark, which we do not exactly profess to comprehend:—"owing to the prejudices of the natives, they seldom consent to undergo an operation, until the powers of the constitution are almost exhausted, and the blood has become quite *corrupted.*"

sometimes be administered. In this case, 78 grains of opium and 27 of morphia in tincture, were given in nine days, before the symptoms yielded. In two of these days about eight grains of morphia were given daily before sleep was induced; and of the opium 18 grains were exhibited in one day, and 39 grains in another. About half the number of dysenteric cases admitted died, chiefly in patients admitted in the advanced stage of the acute or the chronic form of the disease. The case of fatal meningitis supervening in Quotidian, is precisely such an one as we should like to have had a detailed report of—as well as of the quantity of blood abstracted in the first instance, &c. Twenty cases of pneumonia were admitted, of which 13 died 24 hours after admission. The season at which this occurred ought to have been mentioned. Lower down in the page (28) we are told that two of the cases included under the head of Pneumonia died of diseased heart. Let us split the difference, and to make assurance doubly sure, grant that they died of both! We give a short but interesting extract concerning—

"*Spleen Diseases.*—Eight admissions all successfully treated. In one case the spleen was enormously enlarged. This viscus is connected, in a remarkable manner, with the stomach by the vasa brevia, adhesions and ulcerations may have gradually taken place between the two organs, as the patient was suddenly seized with severe vomiting of dark grumous, clotted blood, mixed with shreds of coagulated lymph. This pulpy substance seemed to be the dissolved spleen, and was brought up to the extent of several pounds, after which the viscus was suddenly reduced to its natural size, and the patient left the hospital convalescent. This is supposed to be the only instance known in this part of India, of diseased spleen bursting into the stomach." 29.

From the few cases of ophthalmic affections reported, we infer that the majority went to the Eye Hospital, there being, we believe, such an Institution at Bombay. The notification respecting ulcers is interesting.

| Ulcers | Admitted | Died |
|---|---|---|
| Scorbutic | 16 | |
| Simple | 79 | 2 |
| Sloughing | 11 | 7 |
| Irritable | 3 | |

"One hundred and nine admitted, nine deaths, all occurring in subjects where the disease had become incurable from the state of the general habit, or from the parts becoming so much involved as to prevent amputation. At an earlier stage of the disease, when operations might have been performed with success, the patients could not be persuaded to submit. In some of the cases diarrhœa supervened and carried off the patients. One of these sufferers, an old man, was admitted from the Dhurumsalla. Extensive ulceration of one foot existed with induration and discoloration, the consequence of a leprous diathesis; purging ensued and destroyed the patient. Another individual, having extensive sloughing of the foot and loss of the toes, accompanied with necrosis of the tarsal and metatarsal bones, consented to amputation, but on the table, his courage failing him, he was removed. Scurvy of a most severe character, in this case, supervened, resisting all the remedies, and notwithstanding the greatest attention the patient died, after enduring the greatest suffering, in ten days after the septic symptoms appeared." 30.

The following brief but graphic sketch will give some notion of what one is too often apt to witness in the East.

"In another case, a most horrible object, was taken out of a ditch, left there no doubt to perish; sphacelus of the penis, involving the whole of the scrotum, had taken place; the parts were a perfectly soft gangrenous mass. The patient was pulseless and almost lifeless. The mortified parts were immediately removed; the natural passage of the urethra was discovered, and the accumulated urine drawn off. Yet, notwithstanding the most assiduous employment of ammonia and the diffusible stimuli, the powers of life could not be restored." 31.

The case of strangulated hernia mentioned at p. 32, shews (at least with respect to Natives of India) that one should not despair of the success of an operation even when circumstances appear the most discouraging. *Poisoning* is included under the head of "other Diseases"—and the practice is illustrative of the peculiar morality that "the mild and innocent Hindoo" occasionally exhibits, when he wishes to make free with your property. A London thief will dive his hand into as many of your pockets as he can, and extract the contents. Blackie takes care to make all secure by first stealing your brains, artfully dropping the pounded seeds of Datura in his victim's pots and pans. The recipient, eating of these *hocussed* curries or sweetmeats, gets curiously fuddled at first, playing all sorts of antics, and then falls away into a state of insensibility, when his friend disencumbers him of all his moveables, and then takes French leave. This system is very common all over India—and death sometimes ensues;—but what of that? It is an accident that these Jeremy Didlers of the Tropics are mighty indifferent about, so long as they feel satisfied that dead men tell no tales.

The annual report of H. M. 2nd, or Queen's Royal Regiment is full and able, and abounds in those particulars, the want of which in reports leaves the mind so dissatisfied. It says much for the temperance of the regiment, that during a year there had not been a case of delirium tremens. In noticing the decreasing number of cardiac and aortic diseases, Mr. Hunter remarks—"it was no doubt the frequent and protracted brigade drills of Poona, *aided by the still faulty dress,* which rendered these diseases so remarkably fatal at that station." The same conjoint cause is in frequent, it may be said continual operation, at each of the Presidencies, and is unquestionably productive of much mischief. It is now upwards of a quarter of a century since Dr. James Johnson in his tropical hygiene wished that "the European badge of distinction could be dispensed with at all festivals, public and private, formal, social or domestic, within the torrid zone." This was a philosophical wish, but commandants are not always very philosophical. They rather like the pomp and circumstance of glorious war even in the drawing room, or the ancient hall. It is now upwards of twenty years ago that a pleasant association was put an end to in Calcutta by the despotism of etiquette in dress. It was formed of a number of gentlemen of the different services, and a few professors—the whole rejoicing in the designation of the Amphion Club. Amongst the members was the General Officer commanding at Fort William. The weather in Calcutta *is* sometimes tolerably warm, and no mistake. The members attended the first meeting of the Club ready for action, and dressed in the light habiliments which make oriental heat endurable. Our Major-General's delicate sense of military proprieties was shocked at this. It jarred upon his *amour propre.* The indecorum must be abated. In the interesting history of that eminent self-denying young hero, Jack Horner, we read that

" Jack lov'd mutton pies—
*But* he lov'd to be wise."

Our Martinet loved music, being himself musical also—but he loved regimentals. So unsuspicious of any harm, the good easy man gave an order potential that all military tweedledums and tweedledees, members of the renowned Amphion Club, should always come dressed in full military uniform. Fancy a stout gentleman besworded, besashed and becoated and bebooted playing an obligato on the violin, another blowing a clarinet with a face minacious of apoplexy, and a third wrestling desperately with the double bass! The idea was dreadful—and so with a whir the Amphionites dissolved themselves into thin air and never met in harmony again—but consoled themselves with sundry puns, each exclaiming *am-fie-on-it* at the Martinet's ill-timed zeal for uniformity of costume! This constantly going in harness like a cab-horse may have a very military look, but is very uncomfortable, and certainly injurious to health. Many is the man too, sent to his long home eventually by the system of over-parading, or rather too much parading on days, or at periods of the year, when the men ought to be quietly in their barracks. The Regimental Surgeon, though his hospital may be overflowing in consequence of such over-parading, feels probably like the writhing Prince of Denmark.

" But break my heart for I must hold my tongue."

If commanding officers were to think more about the health of their men, and of their moral happiness than their appearance, on parade or frequency of drill, it were well for the service and the State. Some there are who literally are a curse to all under their authority, keeping every one from the Major down to the Drummer-boy in a continual fret and worry, inimical to good order, discipline and real efficiency, to say nothing of health. How often have valuable lives been hazarded or lost by men being kept hours and hours in the sun, or the rain, perhaps adding to the vain pageantry of a funeral, or awaiting to salute some great man who ought to be above the vanity of exposing men unnecessarily in a trying climate on account of a mere passing reception, a ceremony often more honored in the breach than the observance. Mr. Martin* mentions a circumstance respecting a very distinguished and gallant regiment (H. M. 13th Light Infantry) to which it is probable that, if medical men could always speak out, many a parallel case might be found. " The corps was marched cruelly, because unnecessarily, during the hot season from Nudden to Burhampore. The young soldiers had previously been drilled in the sun thrice a day, so as to greatly injure their healths before quitting Calcutta." The effects we are further told were fatal to a remarkable degree.

In what Mr. Hunter terms cerebral fever, more peculiar to the termination of the hot season, we seem to recognise the same disease as that which came under the observation of Dr. Henderson and Dr. Monat in Bengal—and which afterwards came prominently under the attention of

* Influence on Tropical Climates, &c.

the latter very able and distinguished physician at Madras, and obtained the marked notice of the late Dr. Murray, the lamented Inspector-General of Hospitals. "In this fever"—says Mr. Hunter, "I believe the best practice to be a moderate venesection on admission, 12 or 14 oz. according to the tolerance, age, and constitution of the patient. Large bleedings I conceive to be positively injurious in all the fevers of Deesa, by creating or increasing the congestions." How a free bleeding in this or any other fever should *create* congestion, is a paradox which Mr. Hunter does not explain. We shall, however, perhaps be able to understand his meaning better with Dr. Monat's assistance. Dr. Monat, in the cases of *Insulation* which fell under his notice, remarks that, large bleedings in some instances did no good. "In fact, two individuals became convulsed, and shortly after they were bled, died; and after death, it was found, that, *though the heart was empty, the vessels of the head were loaded with blood.*" Dr. Monat does not fall into the phraseology of Mr. Hunter, and say that this state of the cerebral vessels was *caused* by the bleeding; he knew better, and finding such a state exist notwithstanding the bleeding, he, like an enlightened practitioner as he is, took the hint. He did not leave off bleeding entirely, but made an alteration as to time, and directing cold and cold affusion to the head, bled when re-action took place; or when the vessels of the brain braced by that stimulation, baled back, as it were, the confined wave into the general system. In his remarks on what he calls gastro-enteric or autumnal remittent, Mr. Hunter warns us to have constant reference to the physical signs, since otherwise it is sure to be mistaken for pulmonary disease. "Whether it be from the pain caused by the depression of the diaphragm, I know not, but they refer the seat of it to their chest, which taken with the dyspnœa or panting, is sure to mislead." We are not quite clear that we entirely comprehend our Reporter's balanced remarks on bloodletting. In all except the gastro-enteric, he is persuaded that it is injurious, "yet these cerebral symptoms are so insidious during the first or second exacerbation, that we question whether it is not the safer rule to bleed in every case on admission, to the extent of the tolerance, and particularly with young soldiers of a full habit."—Well, but now comes a damper—"nevertheless it must be admitted that the greater proportion of the deaths by hepatic abscess and dysentery as sequels, have occurred where antiphlogistics, and more particularly bleeding, have been most freely employed." We are free to confess that all this does seem somewhat extraordinary, for supposing the free bleeding and the rest of the antiphlogistic treatment to have been properly and judiciously applied, according to individual exigency and necessity, it no more appears why such sequels should be the result, than depriving a lamp of so much oil, should make it burn twice as long and brightly as it otherwise would. The cases of consumption furnished by Mr. Hunter are full of interest—and he adverts to a species of tuberculous disease among soldiers, that is masked under symptoms of dysentery, and from the frequency of that disease in India, is *constantly mistaken for it.*

No. V. is a series of reports made to the Medical Board on the Climate and Medical Statistics of Upper Scinde. Bowel complaints and fevers as usual, were the prevalent complaints. The season had been one of unprecedented heat. During the day a high wind generally prevailed from the westward, with clouds of dust, while at nightfall it veered to the East. They were in a most open situation, with no shelter save that of the tents. Dr. Owen with probably great justice blames the water. The following table of the range of the Thermometer during five months, is furnished by Mr. Patch, Surgeon, 21st N. I.

| | April. | May. | June. | July. | August. |
|---|---|---|---|---|---|
| Minimum . . . . . | 38 | 53 | 63 | 64 | 62 |
| Maximum . . . . . | 92 | 104 | 107 | 109 | 107 |
| Mean of Minima . . | 58 | 75 | 82 | 83 | 69 |
| Mean of Maxima . . | 76 | 84 | 97 | 103 | 99 |

The temperature, it appears, goes on increasing in a regular manner monthly to July inclusive, but sudden changes are of frequent occurrence, and comparatively cold gusts of wind come down from the ranges of the mountains. There is great increase of fever cases as the temperature diminishes. Mr. Wright, Assistant Surgeon 25th N.I. reporting on the climate of Moostoong, shews fever to be the predominating disease. It was the same at Kelat. The cause, Mr. Wright refers to malaria and solar heat. At Kotra Mr. Babington, Assistant Surgeon of cavalry, reports the climate as exceedingly bad—the number per cent. under medical treatment from 1st Feb. to 31st August 1841, (the best months) being 27 per cent. per mensem, and 112 during the worst. The mortality during four months was 13 out of 175. Respecting Shiakarpore, Assist. Surgeon G. F. Forbes, 8th Regt. N. I. states the worst months to be from Oct. to March—and the number of Sepoys under medical treatment during that time as 36 per cent. per mensem. Deaths none. Assistant Surgeon Jephson reports the climate of Sukkur as very oppressive, and this has been further experienced to be the case in connexion with the operations of Sir C. Napier. The unhealthy season is during Oct. and Nov. while the Indus is falling after the annual inundation. Malaria is, of course, rapidly generated, and to this may we principally look, as the cause of a fever variable in character from the intermittent to the remittent type, the latter often assuming a typhoid form. He considers the climate, however, as healthy for natives. Surgeon Edwards, 23rd N.I. considers the climate (being very dry) as decidedly healthy, save during October and November. The average number of his regiment under medical treatment during the best months, was 6 per cent. and 12 during the worst. The camp ground had been chosen by medical advice, and the result proved how judiciously. Medical officers are too seldom consulted on the site of encamping ground or barracks. This is an unfortunate oversight, and thousands of lives have been the sacrifice. Once let barracks be built, or any line of cantonments in an insalubrious situation, and the mischief is incalculable. On this head we would say most literally to the enquirer at any of the Presidencies and throughout India, si monumentum quæris circumspice. If we might hint a possibility of improving Medical Board returns, perhaps it might be in the form of a note, specifying how long the subscribing medical officer had been in India. This would be an invaluable guide to us reviewers residing at a distance, in forming an estimate of the amount of dependence to be placed on individual experience.

Article VI. gives an account of the Epidemic which prevailed among the men of H. M. 17th Regt. when quartered in Colabah Barracks, after its return from the Affghan campaign, In June 1841, the whole regiment was in barracks at Colabah until September, when the head quarters embarked for Aden, leaving nearly 200 sick at Bombay. As well as we can understand (for Dr. Thomson does not preface his returns with a description of the fever) the disease may be considered to have been a cerebral remittent with great depression of the vital powers, or apparent depression. No question is of greater importance than that of bloodletting in many diseases, but especially in such as these fevers, where it is manifestly one of time, requiring the greatest nicety of decision and discriminative tact on the part of the practitioner. Bloodletting, Dr. Thomson tells us, was tried in some cases, but did not succeed, Let us look a little into this matter. We admit upon the evidence of that experience which every tyro soon acquires without Armstrong's inspirations being required to prove it, that in some cases where there is congestion (more especially in the head) depletion must have a reference to re-action. Case No. 3 is manifestly given "*to illustrate the injurious effects of depletion.*" Here is the case.

" Private Thomas Footed, aged 25 years; in India 2½ years; of a muscular frame; admitted on the 18th June, 1840, with fever of a low type, and with symptoms of congestion of all the organs, was bled to 16 ounces, and had sixty leeches applied to the head, and a blister to the neck; a dose of calomel and antimonial powder and an ounce of wine to be given every second hour.

" *On the 29th the report is,* pulse 125, weak ; no remission of fever; no sleep; bowels open; respirations frequent and labouring; no irregular sound from lungs; great prostration of strength. A blister was applied to the chest, five grains of calomel and two of opium given, and wine continued every second hour.

" 4 P.M. 29. Blister risen well; heart's action and that of lungs increased; pulse 128, small; skin hot and moist; no clammy perspiration.

" 9 P.M. 29th. Is much worse; pulse scarcely perceptible; had slight delirium; skin covered with cold perspiration. Expired at 10 P.M. on the 29th June.

" *Sectio Cadaveris.* Body in good condition and muscular; slight congestion of the lungs and liver; considerable effusion of water on the base of the brain and along the spinal canal."

We have to regret that, in the above case, the state of the face, the eyes, the tongue, and the pulse is not mentioned. We infer that the skin was very hot. We are not told in what position the man was when bled. We are not informed why 16 oz. and no more was abstracted. It could scarcely be from faintness, or collapse, since 60 leeches were forthwith clapped to the head. While depletion is going on with one hand, however, stimulation with wine is carried on with the other. Would not cold affusion have been beneficial in such a case. Dr. Monat, when actual disease of the same nature occurred with sinking of the vital powers, would give a grain of opium to produce re-action and then bleed largely, followed by full doses of calomel and purgatives. The history or rather the abstract of two fatal cases preceding the one quoted, is not sufficiently precise to give us very clear ideas upon the subject. In regard to the first, the duration of the symptoms previous to admission is not stated, nor the state of the bowels. On the 10th of October there was *severe increase* of the fever. Was not the use of the lancet then indicated ? Examination of the body after death shewed serous effusion at the base of the brain, and along the spinal cord, congestion of blood in the internal organs (where ?) of the body. In the second case, the patient is admitted on the 9th of Oct. there is great prostration of the powers of life and slight headache, (p. 116). He gets a large dose of quinine. The remission lasted two hours. No blood had been taken away in the accession—some pain in the abdomen. On the 11th, 60 leeches are applied to the abdomen, but we are not told whether this was at the accession of a paroxysm. Judging from the result we should say it *was not*, but the contrary, since the report on the 12th is "extreme collapse"—and next day death ensues. The post-mortem results much the same as in the case already quoted. The cause of the fever is made sufficiently clear by the following extract. Who built the barracks at Colabah ? On whose advice was the locality chosen ?

"1st. The barracks at Colabah are built almost on a level with the sea. 2nd. The floor is formed of a deliquescent stone, which in damp weather, is always wet, so that it is impossible for the men to keep their feet dry. 3rd. During the rains the water stagnates round the barracks, there being no proper facility for running it away, so that the ground becomes a perfect marsh. 4th. The sea recedes so far as to expose to the sun half a mile of shore covered with slime and marine vegetation; and as the privies are situated here, the smell is often disagreeable." 90.

Mr. Moffat's Report (Art XI.) is an excellent one. He insists upon the propriety of an European regiment, for the first year of service in a Tropical climate, being kept at a healthy station. This has been a matter not sufficiently attended to; and we would refer the reader to Mr. Martin's article "On the Selection and Improvement of Localities for the European Troops." One great desideratum during the prevalence of rainy weather he strongly recommends, and we can testify to its judiciousness, we mean the supply of guard-rooms with stoves, to enable the men to dry their clothes and to warm themselves, after their turn of sentry is over. There is much in this valuable paper that we should like to quote if we could spare the space. Although Mr. Murray's paper on the use and abuse of Calomel contains no new facts, and no information but what may be found in other works,* still it is useful to be able to refer to a document where all the effects may be seen at a glance.

* See Influence of Tropical Climates, p. 281, the work passim, &c.

